# Recent Advances in Metal Organic Frameworks Based Surface Enhanced Raman Scattering Substrates: Synthesis and Applications

**DOI:** 10.3390/molecules26010209

**Published:** 2021-01-03

**Authors:** Panxue Wang, Yan Sun, Xiang Li, Li Wang, Ying Xu, Guoliang Li

**Affiliations:** School of Food and Biological Engineering, Shaanxi University of Science & Technology, Xi’an 710021, China; wangpanxue@sust.edu.cn (P.W.); 1904016@sust.edu.cn (Y.S.); 201604010409@sust.edu.cn (X.L.); 200412101@sust.edu.cn (L.W.); xuying@sust.edu.cn (Y.X.)

**Keywords:** metal-organic frameworks, metallic nanoparticles, nano-composites, applications, food filed, environment field, delivery

## Abstract

Metal-organic frameworks (MOFs) are supramolecular nanomaterials, in which metal ions or clusters are connected by organic ligands to form crystalline lattices with highly ordered periodic porous network structure. MOFs have been widely applied in various fields, such as catalyst, sample preparation, and sensing. In recent years, MOFs based surface enhanced Raman scattering (SERS) substrates have attracted much attention since MOFs can largely improve the performance of metallic SERS substrates toward target enrichment and signal enhancement. MOFs have been exploited in SERS analysis to tackle some challenges that bare metal substrates cannot achieve. Combination of MOFs and SERS improved the sensitivity of traditional SERS analysis and extended the application scope of SERS. With the increasing exploration of MOFs based SERS substrates, there is a great demand to review the advances in these researches. Herein, this review concentrated on summarizing the preparation and applications of MOFs based SERS substrates. Representative researches were discussed to better understand the property of MOFs based SERS substrates. The advantages of MOFs based SERS substrates were highlighted, as well as their limitations. In addition, the challenges, opportunities, and future trends in MOFs based SERS analysis were tentatively discussed.

## 1. Introduction

Surface-enhanced Raman scattering (SERS) is an ultra-sensitive vibrational spectroscopic technique and a versatile analytical tool [1,2,3]. It is an integration of Raman spectroscopy and nanotechnology. Raman spectroscopy gives the fingerprint spectrum of molecule, but is not widely applied due to the inherently weak Raman signals [4,5]. Introduction of noble metal substrates greatly enhanced the Raman signals by 10^4^–10^10^ times, which is beneficial for target characterization and detection [6,7]. SERS has attracted increasing attention in material characterization, analytical science and biomedicine [8,9,10]. There are two well-received theories for this phenomenon, electromagnetic mechanism and chemical enhancement mechanism [1]. In general, electromagnetic enhancement contributes most of the enhancement and it works simultaneously with chemical enhancement. The SERS enhancement effect largely depends on the SERS substrates, normally metal nanoparticles (NPs) or nanoscale roughed surfaces with strong local surface plasmon resonance [11,12,13]. In addition, the analyte should be placed in close proximity to the SERS substrate in order to obtain SERS spectra in high quality. Since the performance of SERS is largely depend on the SERS substrate, it is vital to develop effective SERS substrates for practical applications. Although a variety of SERS substrates have been reported to meet the requirements of reproducibility, stability and sensitivity, more efforts are still needed to overcome the hindrance of offering highly ordered hot spots, selective adsorption and rapid concentration of targets, reducing matrix interference, and tunability for different requirements [14,15,16]. Therefore, exploring SERS substrates with good performance is critically important for promoting SERS development as well as broadening its application scope.

Metal-organic frameworks (MOFs) and composites of MOF and SERS-active metal substrate have been attracting increasing attentions in recent years since these substrates provide additional advantages on stability, target concentration, and selectivity to conventional SERS substrates [17,18]. MOFs have been regarded as an intriguing class of supramolecular nanomaterials, in which metal ions or clusters are connected by organic ligands to form crystalline lattices with highly ordered periodic porous network structures [19,20]. MOFs with large surface area and porous structure exhibit good adsorption capacity and can be used as solid-phase micro-extraction materials for target capture and enrichment [21]. The structure of MOFs is adjustable and diverse, resulting in their broad application in various fields, such as separation [22], storage [23], catalyst [24], sensing [25,26], and biological aspects [27,28]. MOFs also have the feature of good chemical and thermal stability, and can be functionalized by post-modification or post-synthesis [29,30]. MOF based materials have been explored in SERS analysis to tackle some challenges that bare noble metal substrates cannot achieve. For example, many organic molecules exhibit weak affinity to conventional metal SERS substrates and face the challenge of low sensitivity in SERS determination. MOF endows MOF based SERS substrates with improved SERS activity toward gas [31], polycyclic aromatic hydrocarbons (PAHs) [32], and volatile organic compounds (VOCs) [33]. The diverse structure and post-modification of MOF based substrates provide more options for SERS analysis such as rapid target enrichment [34], molecular selectivity [35,36], and signal conversion [37].

It should be noted that some MOFs can contribute extra SERS enhancement and could be used as SERS substrates independently. SERS enhancement effects of MOFs have been first observed on MIL-100 (Al), MIL-100 (Cr), and MIL-101 (Cr) without the help of any other enhancing material [38]. The SERS effect of MOFs was mainly due to the charge transfer effect between the adsorbed molecules and metal oxide clusters in MOFs. In addition, MIL-100 (Fe) has been applied as SERS substrate for the detection of VOCs [39]. The Raman enhancement effect of MIL-100 (Fe) for toluene was up to 10^10^.

**Figure 1:**
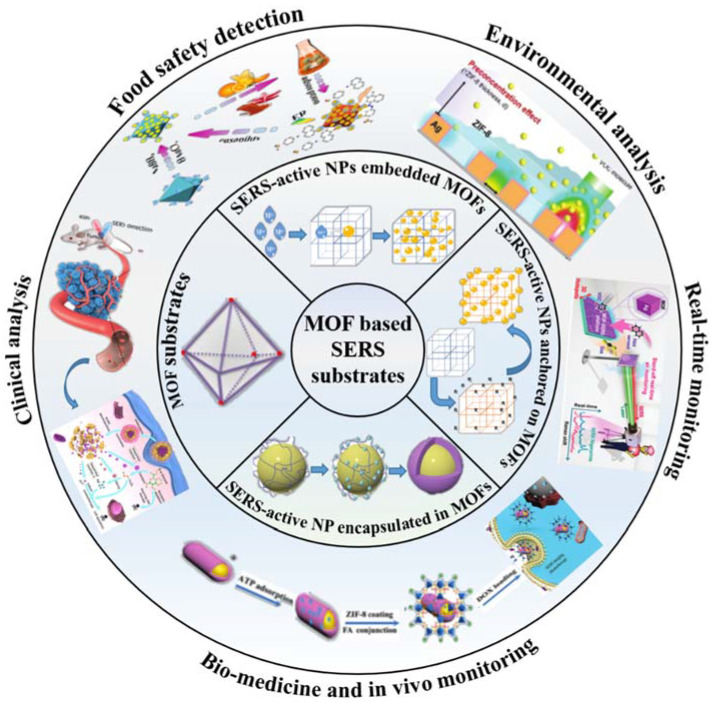
Schematic illustration of the synthesis and applications of MOFs based SERS substrates.

Along with a further mushrooming in the development of MOFs based SERS substrates, there is a great demand to review the recent advances in the emerging MOFs based SERS researches. Herein, this review concentrated on providing detailed information on the preparation and applications of MOFs based SERS substrates (Figure 1). The advantages of using MOF based materials for SERS analysis are highlighted, as well as their limitations. In addition, the challenges and opportunities offered by MOFs based SERS substrates were tentatively discussed. Finally, future trends in the MOFs based SERS analysis are proposed.

## 2. MOF Substrates

SERS effect has been directly observed on some MOFs without the help of classic metal SERS substrates. For example, SERS effects of MIL-100(Al), MIL-100(Cr) and MIL-101(Cr) for methyl orange have been reported by Tsung-Han Yu et al. [38]. The SERS effect was ascribed to the charge transfer effect between the metal clusters in MOFs and the absorbed methyl orange molecules. In addition, MIL-100(Fe) was used as SERS substrate for the detection of VOCs [39]. The MIL-100(Fe) based SERS detection platform showed a detection limit of 2.7 × 10^−2^ µM for toluene. Using MOFs as absorbing and enhancing substrates for VOCs efficiently solved the problem of their shorter adsorption equilibria on noble metal SERS substrates. In addition, MIL-100(Fe) can be extended as a multiplex detection array since it exhibits various binding sites for different targets. For example, the aromatic ligand can bind with VOCs through π-π interaction, and the metal center can bind with small polar molecules by Fe-heteroatomic bond.

Furthermore, MOFs were exploited as SERS substrates with molecular selectivity, which was hard to realize by traditional SERS substrates without functionalization. Hongzhao Sun et al. [40] demonstrated that MOFs could be used as SERS substrates with molecular selectivity owing to their high tailorability. In this study, MOFs based SERS substrates were tailored by controlling the metal center, organic linkers, and framework topologies to match the targets. After optimizing the pore structure and modifying the surface, SERS effect of ZIF-67 for R6G was as high as 10^6^ which was comparable to that of the recently reported semiconductors substrates and the classic noble metal SERS substrates without hot spots. The selective SERS enhancement phenomenon of MOFs was ascribed to the comprehensive effects of several resonances, including charge transfer resonance, molecule resonance, and inter-band resonance.

MOF substrates allow high flexibility and tailorability in design, which are of great importance for specific and selective SERS detection of analytes. In addition, MOF materials with SERS activity offer great opportunities to study the chemical enhancement theory of SERS. However, the SERS effect has only been observed in a few MOFs and efforts are needed to explore more SERS-active MOF materials.

## 3. Nano-Composites of MOFs and SERS-Active Metal Substrates

Except for MOFs with SERS activity, composites of MOF and SERS-active metal substrate have been used in SERS analysis. Combining MOF with metal substrate can result in a synergistic effect between MOF and metal substrate and an improved performance of SERS analysis. Rational design and synthesis of SERS-active NP and MOF composites could be an effective solution to the challenges in current SERS analysis. Depending on the position of SERS-active NPs, composites of SERS-active metal NP and MOF can be classified into three groups, namely SERS-active NPs embedded MOFs, SERS-active NPs anchored on the surface of MOFs, and SERS-active NP encapsulated in MOFs. Synthesis and applications of MOF-based SERS substrates were summarized as below with the highlight of their advantages and limitations.

### 3.1. SERS-Active NPs Embedded MOFs (SERS-Active NPs@ MOFs)

SERS-active NPs embedded MOFs were exploited to solve the challenges of easy aggregation of colloid metal NPs. In this type of composites the porous MOFs not only provide support for SERS-active NPs but also protect them from the outer environment to prevent their migration and over aggregation. The unique structure of porous MOFs provide three dimensional space to accommodate many SERS-active NPs. In addition, MOFs exhibit large surface area and uniform cavities which facilitate the enrichment of target molecules in close proximity to the inner metal NPs. Therefore improved stability and SERS sensitivity were achieved by this type of substrates than conventional metal NPs.

#### 3.1.1. Synthesis

##### Solution Impregnation Method

SERS-active NPs embedded MOF substrates were normally prepared by solution impregnation strategy. Briefly, small sized metal NPs are directly formed inside the as-prepared MOFs and can grow into large sized SERS-active NPs with different size and shape. This strategy is known as a “ship-in-a-bottle” approach and has been widely applied in the preparation of metal NPs embedded MOF substrates [41]. For example, composites of Au NPs embedded MIL-101 were synthesized via in situ growth of Au NPs inside MIL-101 [25]. In this study, the as-prepared MIL-101 was dispersed in aqueous chloroauric acid solution, heated to vigorous boiling, then injected with sodium citrate and kept boiling for 40 min with stirring. After reaction, the hybrids was separated by centrifugation and stored in deionized water. The composites combined the SERS enhancing capability of Au NPs and the adsorption property of MIL-101 and were demonstrated to be highly sensitive to several different analytes.

##### Chemical Vapor Deposition (CVD)

CVD is a solvent free vacuum deposition method. Application of CVD technique to prepare metal NPs embedded MOF substrates was based on the usage of high volatile organometallic precursors [42]. The vapor pressure of gas-phase organometallic precursors propels the entire diffusion process of volatile precursors into the desolvated MOFs cavities. CVD method was first introduced to synthesize metal NPs embedded MOF composites by Fischer’s group [43]. In this study, the freshly prepared MOF-5 was gently dried and used as host matrix to trap [(η^5^-C_5_H_5_)Cu(PMe_3_)] and [(CH_3_)Au(PMe_3_)]. The infiltration process was conducted in a tightly sealed Schlenk-tube under static vacuum (1 atm) condition at 293 K. During the infiltration process the color of MOF-5 changed from light beige to dark red. Then the trapped organometallic precursors were reduced by H_2_ gas, to generate Cu NPs and Au NPs inside MOF-5.

##### Solid Grinding

Similar to CVD method, solid grinding strategy is also a solvent free strategy and in need of gas-phase organometallic precursors. In solid grinding method, the gas-phase organometallic precursors diffuse into the cavities of MOFs during the grinding process and then reduced by H_2_ gas to form metal NPs embedded MOF substrates. This method has been applied in the preparation of various Au NPs embedded MOFs [44,45]. (CH_3_)_2_Au(acac) can be easily diffused into the cavities of MOFs and is a commonly used precursor for growing Au NPs. To make Au NPs embedded MOFs, a desired quantity of (CH_3_)_2_Au(acac) was first mixed with pristine MOF crystals and uniformly ground in an agate morta in air at room temperature. Then the mixture was treated with a flow of 10% (*v*/*v*) H_2_ in N_2_ for 2 h at 120 °C. The generated Au NPs exhibited a nearly uniform morphology with a much smaller size than that produced by CVD method [46].

##### In Situ Growth of MOFs in the Presence of SERS-Active NPs

In situ growth of MOFs in the presence of Au nanorods (NRs) to form hybrids of Au NRs embedded MOFs has been reported [47,48]. For example, Kouta Sugikawa et al. [47] fabricated composites of Au NRs embedded MOF via in situ growth of Zn_4_O(bpdc)_3_ MOF in the presence of 11-mercaptoundecanoic acid modified Au NRs. The embedded Au NRs did not destroy the structure of MOFs or interrupt the adsorption and transfer of molecules into MOFs. The inside Au NRs were partially aggregated and allowed in situ monitoring of molecules diffusion inside MOF by SERS. However, this composites were not suitable for using as substrate in SERS sensors due to their large size and inhomogeneous crystallization. They continued their research on developing Au NRs embedded MOFs in small size. Au NRs embedded cubic MOF-5 hybrids in the size of 400–600 nm were prepared via in situ growth of MOF-5 in the presence of 11-mercaptoundecanoic acid modified Au NRs [48]. Au NRs were mainly incorporated in the middle of the composites and changing the concentration of Au NRs could be used to adjust the size of the composite. The Au NRs@MOF-5 composites exhibited good stability and dispersibility in organic solvents, however their application in aqueous solution was limited since MOF-5 was susceptible to water.

#### 3.1.2. Applications

##### Detection of Chemicals with Low Affinity toward Bare Metal Substrates

In SERS analysis, only targets that can be absorbed onto or close enough to the SERS-active substrates can be effectively enhanced. Therefore, how to make molecules with low affinity toward metal substrates locate at the hot spots of SERS substrates has been a critical point in their SERS analysis. SERS-active metal NPs embedded MOFs offer an exciting opportunity for the detection of molecules with low affinity for metal substrates. For instance, Au NPs embedded MOF-199, Uio-66, and Uio-67 prepared by a facile solution impregnation strategy were used as SERS substrates for the detection of acetamiprid [41]. SERS detection of acetamiprid was difficult since bare metal SERS substrates has low affinity for acetamiprid. However, Au NPs embedded MOF composites exhibited high adsorption capability and excellent SERS activity for acetamiprid due to the π-π interaction between acetamiprid and MOFs and the adsorption property of MOFs. Limit of detections (LODs) of 2.0 × 10^−2^ µM, 9.0 × 10^−3^ µM and 2.0 × 10^−2^ µM were achieved by Au NPs embedded MOF-199, Uio-66, and Uio-67, respectively. The improved SERS sensitivity was ascribed to the outer MOFs shell for providing large surface area and suitable pore size that promoted the adsorption and enrichment of acetamiprid. However, this study only conducted SERS detection of acetamiprid in aqueous solution, further research on stability, sensitivity and reproducibility should be conducted for complex matrices or real samples.

##### Environmental and Clinical Analysis

Water stable MIL-101 embedded with Au NPs was used as SERS substrate for environmental and clinical analysis. For example, Au NPs embedded MIL-101 composites prepared by a solution impregnation strategy were used as SERS substrates for sensitive and quantitative detection of small molecules [25]. The Au NPs embedded MIL-101 composites combined the SERS enhancing capability of Au NPs and the adsorption capacity of MIL-101 and were demonstrated to be highly sensitive to several different analytes. Highly sensitive SERS detection of Rhodamine 6G and benzadine were demonstrated with detection limits of 41.75 fmol and 0.54 fmol respectively. SERS detection of p-phenylenediamine in sewage water and river water was conducted using Au NPs embedded MIL-101 composites as substrate and an improved LOD of 9.208 × 10^−4^ µM was achieved. Application of the synthesized composites in clinical analysis was evaluated by detection of alpha-fetoprotein (AFP) in human serum using a SERS-Enzyme linked immunosorbent assay (ELISA) method based on the benzidine-H_2_O_2_-HRP system. A good linearity in the range of 1.0–130.0 ng/mL with a correlation coefficient of −9938 were observed between the intensity of the characteristic SERS signal at 1197 cm^−1^ versus the concentration of AFP. Moreover, this method was further verified for real sample analysis. The results were in good agreement with that of commercialELISA kit and gave satisfactory recoveries in the range of 79.3–107.3%.

##### Catalytic and Biomedical Applications

Au NPs with plasmonic and catalytic properties provide great opportunities for catalysis and nanomedicine applications. Combining SERS activity and catalytic property of Au NPs allows for real-time monitoring of catalytic reaction and multifunctional biomedical applications. For example, Au NPs embedded MIL-101 with peroxidase activity was prepared by in situ growth of Au NPs inside MIL-101(Cr) and used for in vitro SERS detection of glucose and lactate [49]. The synthesized Au NPs embedded MIL-101 acted as peroxidase mimics and were used to oxidize leucomalachite green which was Raman inactive into Raman active malachite green and enhance the Raman signal of the generated malachite green. In addition, the Au NPs embedded MIL-101 composites were assembled with glucose oxidase and lactate oxidase to produce integrative nanozymes for detecting glucose and lactate in vitro using SERS. The integrative nanozymes exhibited good sensitivity and selectivity in glucose and lactate detection and were applied to monitor changes of glucose and lactate related to physiological and pathological conditions in living brains. Moreover, the composites were suitable for investigating other important biological events, for example glucose metabolism in tumor tissue.

MOFs not only provide support and protection for SERS-active NPs that avoid their migration and aggregation. In addition, the large surface area and porous characters of MOFs endow the composites adsorptive capacity for target enrichment and allow the target distribute in close proximity to the embedded SERS-active NPs. Therefore, improved sensitivity and selectivity in SERS detection were achieved (Table 1). In general, composites of SERS-active NPs embedded MOFs are easy to synthesis and inherit the merits of SERS-active NPs and MOFs. However, the size, quantity and distribution of SERS-active NPs inside the MOFs are hard to control, which are important for generating high quality hot spots for SERS enhancement.

### 3.2. SERS-Active NPs Anchored on the Surface of MOFs (SERS-Active NPs/MOFs)

Metal NPs can be assembled on the surface of MOFs to form SERS-active composites. MOFs in the composite serve as support to fix metal NPs as well as solid-phase adsorption material for target enrichment. MOFs provide large surface area to assemble a great number of SERS-active NPs. It is feasible to generate numerous Raman hot spots on the outer surface of MOFs. Therefore, the generated composites combine the SERS enhancement capability of SERS-active NPs and the adsorption capacity of MOFs, making them excellent SERS substrates for highly sensitive detection of analytes in close proximity to the Raman hot spots domains between the adjacent SERS-active NPs (Table 2).

#### 3.2.1. Synthesis

##### In Situ Synthesis Method

SERS substrates with noble metal NPs anchored on the surface of MOFs can be prepared via an in situ synthesis strategy, namely in situ growth of SERS-active metal NPs on the surface of MOFs. In this approach, MOFs were modified with reducing agents or ions with affinity for metal precursors such as tannic acid [50], dopamine [51], and -NH_2_ [52,53] prior to the synthesis of SERS-active metal NPs. These agents facilitate the growth of SERS-active NPs. For example, a homochiral rod-shaped MOF modified with Br^−^ as ancillary ligand to coordinate metal atoms was selected as support to grow Ag NPs [57]. Br^−^ on MOF can slowly combine with Ag^+^ in the solution to form AgBr on the surface of MOF and the subsequent reduction results in the formation of highly order Ag NPs.

##### Assembling As-Prepared SERS-Active Metal NPs on the Surface of MOFs

In situ synthesis of SERS-active metal NPs on the surface of MOFs is easy to conduct but facing difficulties in controlling the size and distribution of NPs. Different strategies for assembling as-prepared SERS-active metal NPs on the surface of MOFs were reported, such as chemical bond connection [54], electrodeposition [32], and electrostatic affinity [55]. In these methods, the morphology and coverage of SERS-active metal NPs can be well controlled. And high-density SERS-active metal NPs can be assembled to generate abundant hot spots for SERS enhancement. In addition, these methods posed less effect to the structure of MOFs than the in situ synthesis method.

#### 3.2.2. Application

##### Food Safety Detection

The novel SERS substrates with metal NPs anchored on the surface of MOFs have been applied in the detection of illegal colorants, pesticides, and other chemical compounds related to food safety. For example, Leilei Wu et al. [52] developed a versatile Au NPs/UiO-66(NH_2_) based SERS platform for the detection of new coccine and orange II in Mirinda soft drink. The composites performed better than Au NPs in SERS detection and LODs of 6.64 × 10^−1^ µM and 1.668 × 10^−1^ µM were observed for new coccine and orange II. And recoveries ranged from 82.92% to 109.63% were achieved for the detection of new coccine and orange II in Mirinda soft drink. In addition, composites of Au NPs anchored on the surface of NH_2_-MIL-101(Cr) were prepared via an in situ reduction method and were used as SERS substrate for the detection of acid orange II [53]. The developed composites exhibited highly selective adsorption and Raman enhancement capability for orange II. Combined with a portable Raman system, an LOD of 1.527 × 10^−1^ µM was achieved for orange II. Moreover, composites of bimetallic Ag@Au NPs and MIL-101(Fe) were prepared by an in situ reduction method and applied for sensitive SERS detection of thiabendazole (TBZ) in juice [56]. The Ag@Au NPs modified MIL-101(Fe) exhibited reasonable stability and good SERS sensitivity and could selectively adsorb and enhance TBZ under the interference of methyl parathion, parathion, chlorpyrifos, and triazophos. A good linearity in the range of 1.5–75 ppm (R^2^ = 0.986) with an LOD of 50 ppb (2.484 × 10^−1^ µM) were achieved for the SERS detection of TBZ. In addition, analytes could be removed from the composites by using the catalytic activity of MIL-101(Fe) for H_2_O_2_. Therefore, the synthesized composites were reusable.

##### Environmental Analysis

Composites of SERS-active NPs assembled on the surface of MOF can be applied for the SERS detection of environmental pollutants, especially organic molecules such as pesticide residues, aromatic dyes, and PAHs. For example, a reusable Ag NPs/HKUST-1(Cu) composites were prepared via an in situ electrodeposition method and used for the detection of PAHs in sewage water, river water, and seawater [32]. HKUST-1(Cu) was used to provide tunable structures to enable selective loading of Ag NPs as well as to provide large surface area for target enrichment. The polyhedral Ag NPs/HKUST-1(Cu) composites showed high SERS activity for 4-aminothiophenol detection with an LOD of 5 × 10^−10^ M. Moreover, the Ag NPs functionalized HKUST-1(Cu) substrates could be applied for on-site and point-of-care detection of pollutants.

##### Detection of Biomarkers

Water stable MOFs assembled with SERS-active NPs have been applied in the detection of biomarkers in human urine. For example, composites of Ag NPs/MIL-101(Fe) were prepared by an in situ synthesis strategy and applied for highly sensitive SERS determination of dopamine [50]. SERS detection was conducted based on the peroxidase-like activity of MIL-101(Fe) and by using 2,2′-azino-bis (3-ethylbenzthiazoline-6-sulfonic acid) diammonium salt (ABTS) as a Raman reporter. This method exhibited good linearity in the range of 1.054 × 10^−7^–2.108 × 10^−1^ µM (R^2^ = 0.992) with an LOD of 3.2 × 10^−7^ µM. And satisfactory recoveries ranged from 99.8% to 108.0% were achieved for the detection of dopamine in human urine. In addition, composites of Ag NPs/MIL-101(Cr) with positive charge could enrich folic acid by electrostatic interaction and allowed the captured folic acid sat close to the anchored Ag NPs, resulting in greatly enhanced SERS sensitivity [51]. A good linearity in the range of 0.5–25 μM (R^2^ = 0.990) was observed with a calculated LOD of 0.3 ± 0.02 μM. The developed method showed good accuracy and reliability in the detection of folic acid in human urine. Moreover, MOFs assembled with SERS-active NPs can be applied in SERS immunosensors for selective detection of specific biomarkers. For example, composites of Au tetrapods (TPs) anchored on the surface of IRMOF-3 were prepared based on the covalent bond between amino group and Au and were used for the SERS detection of N-terminal pro-brain natriuretic peptide (NT-proBNP), which was considered as a specific biomarker of heart failure [54]. A SERS immunosensor with CoFe2O4@AuNPs@Ab1 magnetic substrate as capture probe and IRMOF-3@AuTPs@TB@Ab2 composite as SERE tag was designed for detecting NT-proBNP. An LOD of 0.75 fg/mL was achieved by this method. IRMOF-3@AuTPs@TB contributed to the improved sensitivity of the SERS immunoassay due to the large surface area of IRMOF-3 and abundant hot spots generated by Au TPs.

##### Bio-Medicine and In Vivo Monitoring

Cancer is a major threat to human health. And a large number of therapies have been explored to combat it. MOF based SERS substrates have been explored in the design of versatile nanocarriers for cancer diagnosis and therapy. For example, a degradable nanoreactor was constructed by assembling Ag NP@4-mercaptobenzonitrile (MBN) SERS tags on the surface of ZIF8@Glucose oxidase (GOx) and was used for synergistic cancer therapy and simultaneous glucose monitoring by SERS [55]. This nanoreactor can be activated by tumor microenvironment to start the catalytic enhanced metal ion poisoning and starvation synergistic therapy and to be used as SERS substrate for monitoring of cellular glucose level. The Ag NPs functionalized ZIF8 composites showed a high loading of GOx to ~55 wt%, and the encapsulation strategy protected GOx from enzyme mediated degradation. In addition, GOx activity and glucose level could be monitored by SERS. Moreover, the developed nanoreactor was biodegradable thus avoided the side effects and accumulation in mice with tumor. However, this nanoreactor has only been applied in mouse and still facing limitations for clinical use.

SERS substrates with metal NPs anchored on the surface of MOFs have the advantages of excellent SERS enhancement and selective target enrichment, making these substrates attractive for ultrasensitive SERS detection. The difficulty is how to efficiently assemble SERS-active NPs while maintaining the unique structure of MOFs.

### 3.3. SERS-Active NP Encapsulated in MOFs (SERS-Active NP@MOFs)

Composite of MOF encapsulated single metal NP possesses a well-defined core-shell or sandwich-type structure. The size and shape of the inner metal NP core and the thickness of the outer MOF shell can be easily adjusted. The outer MOF shell protects the inner metal NP from oxidation and erosion and endows the composite adsorptive property for target enrichment. Encapsulating metal NPs within MOFs has been attracting increasing attention in recent years. A large number of MOF encapsulated SERS-active NP composites have been prepared and applied as novel multifunctional substrates for SERS analysis (Table 3).

#### 3.3.1. Synthesis

##### Seed Growth Method

Seed growth method is usually used in the preparation of MOFs encapsulated metal NP composites. In this method, SERS-active NPs are synthesized first and then encapsulated by MOFs. This method can be used to assemble controllable thickness of MOFs shell on the surface of SERS-active NP and is known as the “bottle-around-ship” strategy. Advantages of this strategy are that the composition, size, and shape of the SERS-active NPs can be well designed and controlled. To facilitate MOFs coating, “binder agents” such as polyvinylpyrrolidone (PVP), hexadecyl trimethylammonium bromide (CTAB), and poly(ethylene glycol) (PEG) are introduced to functionalize the SERS-active NPs [66]. For example, quaternary ammonium surfactants, such as CTAB can promote the interaction between SERS-active NPs and ZIF-8. Quaternary ammonium surfactants are widely used as stabilizing agents for colloid metal NPs. In addition, they have been proved to play critical roles in promoting the growth of ZIF-8 in water with different sizes and shapes. Therefore, quaternary ammonium surfactants have been used to promote the interaction between SERS-active NPs and ZIF-8 [67]. The thickness of the ZIF-8 shell can be adjusted by changing the concentration of quaternary ammonium surfactants. And precise control of the quaternary ammonium concentration in the reaction mixture was needed for successful growth of ZIF-8 on the surface of single metal NP.

##### One-Pot Method

The core-shell MOF encapsulated SERS-active NP composites also can be prepared by a one-pot method. In this method the precursors for SERS-active NP and MOF were directly mixed, and then incubated to form composites of MOFs encapsulated single SERS-active NP. For example, core shell AuNP@MOF-74 composites were synthesized by mixing precursors for Au and MOF-74, DMF, PVP, and ethanol together and then reacted at 120 °C for 3 h [58]. In this study, HAuCl_4_ was first reduced by DMF to form Au NPs, then MOF-5 started to grow on the surface of PVP coated Au NP to form AuNP@MOF-5 core-shell composites. The products were separated by centrifugation, washed with methanol, and dried in vacuum. The diameter of Au NP core and the thickness of the outer MOF shell could be adjusted by changing the concentration of Au precursors. The prepared composites exhibited good SERS activity for 4-nitrothiophenol. And a linear relationship in the range of 0.10–10 µM with an LOD of 6.9 × 10^−2^ µM were observed in the SERS detection of 4-nitrothiophenol.

#### 3.3.2. Applications

##### Food Safety Detection

Similar with other types of MOFs based SERS substrates, MOFs encapsulated SERS-active NP composites can be applied for the detection of pesticides [59] and dyes [60]. However, it is difficult to form effective hot-spots by single metal NP core. Efforts have been made to improve the SERS activity of the composites [61,62,68]. For example, multifunctional SERS-active Fe_3_O_4_@Au@MIL-100(Fe) magnetic composites were developed for sensitive detection of dyes and pesticides [62]. In this study, the multifunctional Fe_3_O_4_@Au@MIL-100(Fe) composites were prepared by a three-step method and used for sensitive SERS detection and quantification of malachite green (MG) and thiram. The integrated composites combined the advantages of magnetic separation, SERS enhancement, and target enrichment, and exhibited improved sensitivity for MG and thiram with LODs of 4.4 × 10^−3^ µM and 1.5 × 10^−2^ µM respectively. In addition, Xiaowei Ma et al. [68] prepared Fe_3_O_4_@Au@MIL-100(Fe) magnetic composites for the SERS detection of cationic dyes. The outer MIL-100(Fe) shell endowed selective adsorption of targets and made them locate at the hot spots of the inner Au NPs. Therefore ultrasensitive SERS detection of cationic dyes was established with an LOD of 1 × 10^−3^ µM for MG.

##### Detection of Gas and VOCs

MOFs encapsulated SERS-active NP composites offer exciting opportunities for SERS monitoring and detection of gas and VOCs, which are hard to achieve by bare metal substrates [33,69]. Various ZIF-8 encapsulated SERS-active NP composites were developed for these purposes. For instance, an Au@ZIF-8 array was used for SERS detection of toxic VOCs [63]. The Au@ZIF-8 arrays showed good reproducibility and SERS sensitivity toward VOCs with an LOD of 1.085 × 10^3^ µM for toluene. In addition, ZIF-8 coated Au@Ag nanocubes has been used to fabricate a Polydimethylsiloxane (PDMS) microfluidic chip for multiplex gas sensing [31]. The Au@Ag@ZIF-8 nanoparticles were modified on the side walls of prism and SERS signal of ZIF-8 was served as an internal reference. Gas sensing ability of the developed SERS array was investigated using aldehyde as representative with an equal LOD of 9.423 × 10^−3^ µM was obtained for benzaldehyde and 3-ethylbenzaldehyde. Moreover, Phan-Quang et al. [64] developed an Ag@ZIF-8 nanoparticles based SERS platform for real time stand-off multiplexed monitoring of airborne molecules. The developed SERS method allowed rapid quantitative detection of aerosolized chemicals with LODs down to ppb levels at a distance of 2–10 m.

##### Real-Time Monitoring of Catalytic Activity

MOFs have been explored in the catalysis field owing to their excellent catalytic properties and unique structures. MOF derivatives inherit the catalytic activity from MOF [70,71]. In combination with SERS, MOFs encapsulated SERS-active NP composites can realize real-time monitoring of catalytic reactions. For example, Xiaowei Ma et al. [37] developed Fe_3_O_4_@Au@MIL-100(Fe) magnetic composites for in situ monitoring of the catalytic oxidation of 3,3′,5,5′-tetramethylbenzidine (TMB) by H_2_O_2_. The Fe_3_O_4_@Au@MIL-100(Fe) magnetic composites inherited the peroxidase-like activity of MIL-100(Fe) and good SERS activity of Au NPs and could be used to monitor the peroxidase-like reaction and to detect the concentration of H_2_O_2_ by SERS simultaneously.

##### Drug Carriers

Composites of aqueous stable MOFs encapsulated SERS-active NP have been investigated for multifunctional SERS imaging and being used as drug carriers for therapy. For example, novel SERS probes consisted of ZIF-8 coated individually core shell Au@Ag NR and Raman reporters were used for in vitro imaging and multiplex bio-detection [72]. The Au@Ag NR core was used to enhance the Raman signal and the ZIF-8 shell was used to trap the Raman reporters and load antibodies. Capability of the newly developed SERS tags were verified by SERS detection of cell surface receptors EGFR and CD4. The modular assemble strategy is highly versatile and can be applied as a universal approach for developing MOFs based tags for bio-sensing and bio-detection. In addition, ZIF-8 encapsulated Au nanostar composites were used for drug delivery [73]. In this study, an amphiphilic polymer was introduced to stabilize the composites and protect the loaded cargo from leaking into aqueous media or living cells. The composites showed a high loading of bisbenzimide and could be activated by near-IR light to release the cargo. The loading and releasing process were monitored by SERS. Moreover, Au NP@Cu_3_(BTC)_2_ composites were used for loading drug and in vivo SERS imaging [65]. Cu_3_(BTC)_2_ shell was used to load drugs and provided sites for binding aptamers. The Au NP core acted as a photothermal agent for destructing cancer cells and accelerating drug release and as a SERS substrate for enhancing Raman signal of 4-MBA. The composites showed good biocompatibility, high drug loading capacity (57% for doxorubicin), effective photothermal activity, and SERS enhancement and could be applied for tracking cells and synergistic chemo-photothermal therapy of tumor.

Noble metal NPs encapsulated in MOFs show higher stability than colloidal NPs under different conditions. The outer MOF shell not only stabilize the inner metal NP, but also selectively and efficiently enrich targets. The main challenge facing this type of hybrids is how to generate numerous hot spots with good SERS enhancement.

## 4. Conclusions and Future Perspectives

In this review, we have reviewed the recent advances in the synthesis and applications of MOFs based SERS substrates with the highlight of their advantages and disadvantages (Table 4). MOFs with unique structures and properties provide additional advantages and solutions to current SERS analysis. The large surface area and uniform porous structure of MOFs allow rapid, efficient, and selective target separation and enrichment. MOF materials with SERS activity offer great opportunities for detecting analytes with molecular selectivity and studying the chemical enhancement theory of SERS. Composites of MOFs and SERS-active metal NPs exhibited better stability and adsorption property than bare metal NPs. MOFs based SERS analysis showed improved sensitivity, stability, and reproducibility owing to the unique structure and good chemical and thermal stability of MOFs. In addition, MOFs based SERS could be applied in the analysis of gas, VOCs, PAHs, and molecules with low affinity toward bare metal NPs, which were difficult to achieve by conventional SERS analysis. Therefore, MOFs based SERS analysis showed better performance and broader application range than conventional SERS analysis.

However, MOFs based SERS analysis is still facing some challenges. First, only a limited number of MOFs with SERS activity have been reported. And more efforts are needed in revealing the enhancement mechanism of MOFs and exploring more MOF materials with SERS enhancement property. Second, it is difficult to create numerous hot spots in MOFs based SERS substrates while maintaining the structure of MOFs. Third, large-scale synthesis of MOFs based SERS substrates with uniform and consistent structure is extremely challenging. Finally, identification and explanation of SERS signals in biological analysis are difficult. Despite the above challenges, MOFs based substrates offer additional advantages and great opportunities for SERS analysis. And with increasing attentions have been focused on this field, these challenges would be solved in multiple ways. For example, coating MOFs onto SERS substrates with well-defined hot spot arrays will help to improve the SERS enhancement performance. Choosing Raman reporters with characteristic peaks located at the cellular Raman silent region (1800–2800 cm^−1^), such as alkyne tags, will improve the sensitivity of SERS for bio-analysis as well as simplify the process of spectral data identification and explanation.

## Figures and Tables

**Table 1:** Application of SERS-active NPs embedded MOF composites in SERS analysis.

Type	MOF Based Substrates	Synthesis	Target	LOD (μM)	Matrices	Reference
NPs@MOF	AuNPs/MOF-199, AuNPs/Uio-66, and AuNPs/Uio-67	Solution impregnation method	Acetamiprid	2 × 10^−2^, 9.0 × 10^−3^, and 2.0 × 10^−2^	-	[41]
NPs@MOF	AuNPs/MIL-101	Solution impregnation method	Benzadine	9.208 × 10^−4^	Environmental water, serum	[25]
NPs@MOF	AuNPs@MIL-101	Solution impregnation method	Glucose, lactate	4.2, 5.0	-	[49]

**Table 2:** Application of composites of SERS-active NPs anchored on the surface of MOFs in SERS.

Type	MOF Based Substrates	Synthesis	Target	LOD (μM)	Matrices	Reference
NPs anchored on MOF	Ag NPs/MIL-101 (Fe)	In situ synthesis	Dopamine	3.2 × 10^−7^	Urine	[50]
NPs anchored on MOF	Ag NPs/MIL-101(Cr)	In situ synthesis	Folic acid	0.3 ± 0.02	Urine	[51]
NPs anchored on MOF	Au NPs/UiO-66(NH_2_)	In situ synthesis	New coccine, orange II	0.664, 0.1668	Soft drink, paprika	[52]
NPs anchored on MOF	Au NPs/NH_2_-MIL-101(Cr)	In situ synthesis	Acid orange II	0.1527	Orange juice, Chili powder	[53]
NPs anchored on MOF	Au TPs/IRMOF-3	Covalent bond	NT-proBNP	0.75 fg/mL	-	[54]
NPs anchored on MOF	Ag NPs/HKUST-1(Cu)	Electrodeposition	PAHs	1.5 × 10^−4^ to 2 × 10^−2^	Environmental water	[32]
NPs anchored on MOF	Ag NPs/ZIF8	Electrostatic affinity	Glucose	-	Cell	[55]
NPs anchored on MOF	Ag@Au NPs/Mil-101(Fe)	In situ synthesis	Thiabendazole	0.2484	Juice	[56]

**Table 3:** Application of SERS-active NP encapsulated in MOF composites in SERS analysis.

Type	MOF Based Substrates	Synthesis	Target	LOD (μM)	Matrices	Reference
NP@MOF	Au NP@MOF-74	One-pot method	4-nitrothiophenol	6.9 × 10^−2^	-	[58]
NP@MOF	Ag NP@ZIF8	Seed growth method	Crystal violet	10^−2^	ppb level	[59]
NP@MOF	Au NP@MIL-100(Fe)	step-by-step method	MG	8.0 × 10^−3^	Aquaculture water, fish	[60]
NP@MOF	Au NP@MOF-5	Seed growth method	Organophosphorus pesticides	10^−8^	Soil	[61]
NP@MOF	Fe_3_O_4_@Au NPs @MIL-100(Fe)	Three-step method	MG, thiram	4.4 × 10^−3^, 1.5 × 10^−2^	Water	[62]
NP@MOF	Ag NP@ZIF8	Seed growth method	Benzene	6.917 × 10^3^	-	[33]
NP@MOF	Au NP@ZIF8	Seed growth method	Toluene	1.085 × 10^3^	-	[63]
NP@MOF	Au@Ag NP@ZIF8	Seed growth method	Benzaldehyde, 3-ethylbenzaldehyde	9.423 × 10^−3^	-	[31]
NP@MOF	Ag NP@ZIF8	Seed growth method	PAHs, CO_2_	-	Air/gaseous environment	[64]
NP@MOF	Au NP@Cu_3_(BTC)_2_	step-by-step method	Load drug	-	Cell	[65]

**Table 4:** Summary of the advantages and disadvantages of MOFs based SERS substrates.

Type	Advantages	Disadvantages
MOFs	Diverse structure, molecular selectivity, good for mechanism research	Numbers are limited
SERS-active NPs embedded in MOFs	Easy to synthesis	The morphology, quantity, and location of NPs inside MOFs are hard to control, the structure of MOFs might be destroyed
SERS-active NPs anchored on MOFs	Good SERS performance, easy to synthesis	Difficult to maintaining the structure of MOFs
SERS-active NP encapsulated in MOFs	Good stability, selectivity, and adsorption capacity	Weak SERS enhancement capacity

## Abbreviations

AFP	Alpha-fetoprotein
CTAB	Hexadecyl trimethylammonium bromide
ELISA	Enzyme linked immunosorbent assay
GOx	Glucose oxidase
LOD	Limit of detection
MBN	4-mercaptobenzonitrile
MG	Malachite green
MOF	Metal–organic framework
NP	Nanoparticle
NR	Nanorod
NT-proBNP	N-terminal pro-brain natriuretic peptide
PAH	Polycyclic aromatic hydrocarbon
PDMS	Polydimethylsiloxane
PEG	Poly(ethylene glycol)
PVP	Polyvinylpyrrolidone
SERS	Surface-enhanced Raman scattering
TBZ	Thiabendazole
TMB	3,3′,5,5′-tetramethylbenzidine
TP	Tetrapod
VOC	Volatile organic compound
